# POPKI: Modeling Surprise in a Physically-Grounded Joint Inference of Observation, Preference, and Knowledge

**DOI:** 10.1162/OPMI.a.375

**Published:** 2026-07-17

**Authors:** Harry Chen, Karen Chung, Abhishek Bhandwaldar, Joshua B. Tenenbaum, Tomer D. Ullman, Tianmin Shu

**Affiliations:** Department of Brain & Cognitive Sciences, Massachusetts Institute of Technology, Cambridge, MA, USA; Department of Electrical Engineering & Computer Science, Massachusetts Institute of Technology, Cambridge, MA, USA; MIT-IBM Watson AI Lab, Massachusetts Institute of Technology, Cambridge, MA, USA; Department of Psychology, Harvard University, Cambridge, MA, USA; Department of Computer Science & Department of Cognitive Science, Johns Hopkins University, Baltimore, MD, USA

**Keywords:** intuitive psychology, perceptual access, knowledge, Bayesian inverse planning, violation of expectation

## Abstract

People naturally take into account others’ limited perceptual access when attributing mental states such as preferences and knowledge. This ability is central to intuitive psychology, and even infants recognize when others have only partial observability. Understanding how mental state attributions adapt under limited perceptual access is thus crucial for advancing models of intuitive psychology. Here, we introduce POPKI (Physically-grounded Observation, Preference, and Knowledge Inference), a Bayesian inverse-planning framework designed to model graded surprise based on agents’ inferred preferences and knowledge in visually constrained 3D environments. Building on and expanding the AGENT dataset, we develop new scenarios that test how partial observability shapes judgments of surprise. Despite the tremendous progress of state-of-the-art vision-language models (VLMs), our experiments show that they fail to capture the nuances of perceptual access when judging surprise. POPKI, in contrast, offers a principled approach to modeling human-like uncertainty under partial observability and closely replicates human surprise patterns. It provides a foundation for advancing psychological reasoning models and enhancing human-AI interaction.

## INTRODUCTION

Theory of Mind (ToM)—the capacity to attribute beliefs, desires, and intentions to oneself and others—forms the foundation of human social cognition (Premack & Woodruff, 1978). It enables us to predict behavior in dynamic, ambiguous settings by reasoning about others’ mental states, including what they can see, what they know, and what they want (Tomasello, 1999). Beyond its significance in developmental and cognitive science, ToM has emerged as a crucial goal in artificial intelligence (AI), particularly for systems designed to interact naturally and safely with humans (Dennett, 1987).

A fundamental, first-principles requirement of ToM is that mental state inference is inherently grounded in physical reasoning. To understand what another person *wants*, an observer must represent the physical costs and rewards associated with possible courses of action. To understand what another person *knows*, the observer must reason about what information that person could have perceived (i.e., which objects were visible given the geometry of the environment, which were occluded by walls or obstacles, and what the agent’s line of sight permitted). To understand whether another person is in *danger*, the observer must simulate the physical consequences of their actions going forward. In short, attributing mental states requires grounded representations of the physical scene in which an agent acts. Recent theoretical and empirical work has articulated this dependence explicitly, arguing that naive psychology (the capacity to reason about minds) directly calls upon naive physics (the capacity to reason about objects, forces, and spatial relations) rather than operating as a fully autonomous system (Liu et al., 2026). This interdependence is not unique to sophisticated adult reasoning; it is detectable from the first months of life, when infants already integrate physical constraints on action into their inferences about agents’ goals, preferences, and knowledge (Luo & Baillargeon, 2007).

This first-principles perspective motivates the central problem we address: how should mental state attributions change when an agent’s *perceptual access* to the physical world is limited? If ToM is grounded in physical scene representations, then incomplete physical information—arising from occlusion, obstruction, or gaps in temporal visibility—must propagate systematically into uncertainty about mental states. An agent who never saw one of two available objects cannot have formed a preference about it. An agent whose view was obscured midway through familiarization may be acting on a partially decayed memory of what they once saw. Capturing these dynamics requires a model that explicitly represents the interface between the physical scene and the agent’s mental states, rather than treating them as separable inference problems.

Recent progress in cognitive modeling has yielded several influential computational frameworks for ToM. A landmark example is the Bayesian Theory of Mind (BToM) model introduced by Baker et al. (2017), which uses inverse planning in partially observable environments to infer beliefs, desires, and percepts based on agents’ actions in 2D spatial settings. This model was a major step forward, jointly modeling agents’ perceptual access and planning under uncertainty, and demonstrated strong alignment with human judgments across a range of scenarios.

The BToM model was developed in a controlled 2D setting—a deliberate and productive simplification that allowed the core questions about belief and desire inference to be isolated and rigorously tested. The natural next step, which our work takes, is to ask how such a framework extends to richer physical contexts: specifically, how does 3D visual occlusion and dynamic scene geometry shape what an agent knows, and how does that knowledge feed back into the surprise an observer feels when watching the agent act? These questions about physical and perceptual grounding could not easily be addressed with the tools and computational infrastructure available a decade ago—advances in probabilistic programming, physics engines, and 3D rendering now make it tractable.

Our work therefore takes BToM as a foundation and extends it along two axes that the original framing set aside: the full geometric complexity of 3D line-of-sight and occlusion, and graded, probabilistic inference over the agent’s evolving knowledge state rather than a discretized hypothesis space. We also benchmark against current vision-language models (VLMs), such as Gemini 2.5 Pro and GPT-4o, which—despite their strong performance on multimodal reasoning tasks like image captioning and visual question answering (Comanici et al., 2025; OpenAI et al., 2024)—lack any structured representation of what an agent can or cannot see. As we demonstrate, this limitation leads them to fail on tasks requiring ToM-style reasoning under conditions of limited perceptual access: they do not accurately infer how being unable to see an event or object should constrain an agent’s beliefs, preferences, or future actions.

This shortcoming arises not from lack of data or training scale, but from an architectural blind spot: VLMs lack explicit models of what agents can and cannot see, and how perceptual history constrains belief updating (Lee et al., 2025). They often conflate their own global view of the scene with the partial, egocentric view available to the agent in question (Gao et al., 2025; Wang et al., 2026). Moreover, unlike inverse planning models, VLMs do not simulate agentic decision-making under uncertainty, nor do they distinguish between knowledge, belief, and desire in the structured, causal way that human observers do (Chen et al., 2024).

To address these limitations, we introduce AGENT+, an extension of the AGENT dataset designed to evaluate ToM reasoning under limited perceptual access in 3D spatial environments. Whereas earlier datasets, including the original AGENT and BToM scenarios, relied on simplified occlusions and discrete agent-environment interactions, AGENT+ introduces more naturalistic trials with complex visual occlusion, dynamic object movement, and temporally extended episodes. These trials test whether AI models can track an agent’s perceptual exposure to events and reason about what the agent does or does not know based on that exposure.

To model an agent’s surprise in these scenarios, we propose POPKI (Physically-grounded Observation, Preference, and Knowledge Inference), a novel Bayesian inverse-planning model that integrates 3D perceptual tracking and preference reasoning with belief state updates. POPKI explicitly simulates what each agent has seen (or missed), and uses this to compute graded predictions of belief, surprise, and behavior in uncertain environments. Unlike previous models that assume full observability or static world states, POPKI can handle belief divergence due to occlusion, 3D reasoning, and time-dependent physical interactions.

While Baker et al. (2017) demonstrated that inverse planning under partial observability can account for belief and desire inferences in controlled environments, our work highlights that real-world ToM demands richer visual and physical simulation, explicit 3D modeling of vision, and continual belief updating grounded in physical scene dynamics. In this way, AGENT+ and POPKI represent a significant step toward bridging symbolic cognitive modeling with the physical and perceptual richness of embodied AI.

The remainder of this paper is organized as follows. Computational Modeling section introduces the AGENT+ dataset, outlining its design principles and extensions beyond prior benchmarks. Methods describes our experimental methodology, including both the human behavioral study and the computational formulation of POPKI. Baselines section reports our empirical findings, comparing human surprise judgments with the performance of vision–language models and our proposed approach. Finally, Results and Discussion section discusses the broader implications of these results for building AI systems that exhibit intuitive psychological reasoning under realistic constraints.

## DATASET

### AGENT and AGENT+ Datasets

AGENT (Action and Goal-based Evaluation for Theory of Mind) is a benchmark designed to evaluate ToM reasoning in machine agents (Shu et al., 2021). It assesses whether computational models can infer an agent’s goals, preferences, and efficiency in structured environments. The benchmark consists of animated scenarios where an agent interacts with objects, navigates obstacles, and selects goals based on inferred preferences. Models are evaluated on their ability to predict rational action planning and deviations from optimal behavior, with human ratings providing ground-truth data for comparison. However, AGENT assumes full perceptual access, meaning it does not test whether models can infer an agent’s beliefs under limited observability.

**Figure 1.**
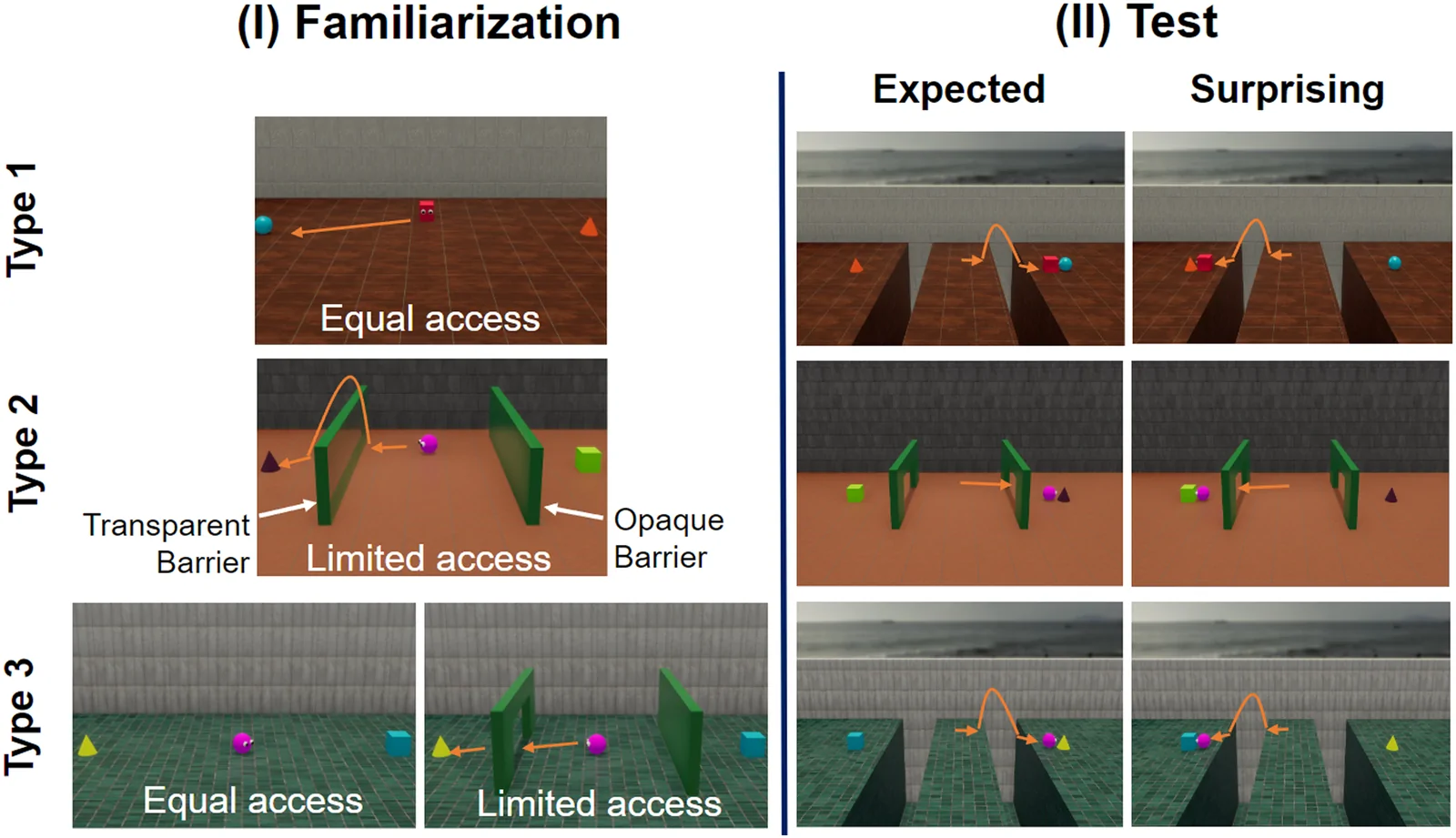
**Overview of the perceptual access trials.** Each trial consists of a *familiarization video* (left), in which an agent (colored block with eyes) navigates a 3D environment containing two goal objects separated by barriers, followed by a *test video* (right) in which the agent chooses between the two objects with both now fully visible. Type 1 models an agent with full knowledge of both objects. Type 2 models partial knowledge, and Type 3 models obstructed vision. One of the barriers in the Type 2 familiarization video is transparent. Two frames are shown for Type 3, as the agent initially sees both objects before the barriers drop.

To address this limitation, we introduce AGENT+, which explicitly incorporates perceptual access constraints. AGENT+ expands the dataset by introducing trials where an agent’s knowledge state depends on their visual exposure to events. For example, an agent may see only one object when two objects exist due to an opaque obstruction, requiring models to infer partial perceptual access. AGENT+ consists of three additional trial types: Type 1 (equal perceptual access), Type 2 (limited perceptual access), and Type 3 (initial equal perceptual access followed by restriction), each designed to test how models interpret behavior under varying perceptual constraints (Figure 1).

Each trial begins with a familiarization video in which an agent demonstrates a preference for a particular object by repeatedly approaching it. This is followed by a test video where the agent selects either the same object or a different one. As in Luo and Baillargeon (2007), familiarization establishes the agent’s preferences, while test trials introduce an unexpected or surprising choice depending on the agent’s perceptual access.

### AGENT+ Perceptual Access Scenarios

1. **Type 1: Full Knowledge (Equal Perceptual Access)**—The agent sees both objects during familiarization. Because the agent has full information, its decision to repeatedly approach one object over the other is strong evidence of a genuine preference. At test, when both objects are again visible, a rational agent with a known preference should approach the same preferred object. Observers and POPKI should therefore *strongly expect* this choice and be *highly surprised* if the agent approaches the other object instead. This condition serves as a baseline for clear preference inference without perceptual constraints.
2. **Type 2: Partial Knowledge (Limited Perceptual Access)**—The agent sees only one object during familiarization due to occlusion by an opaque barrier. Crucially, the agent’s choice to approach that object is *not* freely chosen evidence of a genuine preference: the agent simply had no knowledge that a second object existed, making its behavior constrained rather than expressive. At test, when both objects are visible for the first time, either choice is consistent with the agent’s preferences—the agent may genuinely prefer the familiar object, or it may prefer the newly visible one (which it could not have signaled before). Observers and POPKI should therefore be *less certain* of what the agent will do, and should show *reduced surprise* regardless of which object is ultimately approached. This condition tests whether models appropriately discount constrained choices when inferring preferences.
3. **Type 3: Obstructed Vision (Initial Access with Restriction)**—The agent sees both objects at the start of familiarization but one is later occluded by a barrier. The agent therefore has some knowledge of both objects (from the initial exposure), but its most recent behavior is based on the restricted view. At test, its initial choice carries preference signal, but with additional uncertainty due to the subsequent occlusion and potential memory decay. This condition tests memory-based preference inference and predicts intermediate surprise levels between Type 1 and Type 2.

In all test videos, both objects are fully visible, so any variation in observers’ surprise must arise from differences in the prior perceptual access established during familiarization.

## COMPUTATIONAL MODELING

### Model Overview

POPKI (Physically-grounded Observation, Preference, and Knowledge Inference) jointly infers an agent’s preferences, knowledge, and perceptual access within a probabilistic generative framework.

The core challenge is that an observer watching a video must simultaneously reason about *what the agent can see* (a function of 3D scene geometry), *what the agent knows* (accumulated over time from what it has seen), and *what the agent prefers* (inferred from its actions). These three quantities are interdependent and latent as they are not directly observable. Capturing their joint uncertainty and updating all three as new observations arrive calls for principled probabilistic inference. POPKI is therefore implemented in the probabilistic programming language Gen (Cusumano-Towner et al., 2019), which provides infrastructure for defining generative models over latent variables and running efficient inference algorithms (specifically, Sequential Monte Carlo; see Inference and Surprise Estimation section).

POPKI also uses PyBullet (Coumans & Bai, 2016–2019) as its physics engine and Rapidly-exploring Random Tree star (RRT*) (Karaman et al., 2011) for planning. RRT* is a sampling-based motion planning algorithm that finds near-optimal collision-free paths through 3D space—it generates the trajectories an agent would plausibly take to reach a goal. Together these two components ground the agent’s cognitive states in the physical structure of the scene.

The original AGENT paper (Shu et al., 2021) uses the BIPaCK (Bayesian Inverse Planning with Costs and Knowledge) model, which integrates a physics engine with deterministic planning to simulate agent actions and applies Bayesian inverse planning to infer preferences from observed behavior. It accounts for both action costs and object rewards, allowing it to model rational choice by comparing simulated optimal plans with actual actions.

POPKI extends BIPaCK in several key ways. While BIPaCK integrates physics simulation and deterministic planning to infer preferences and costs under the assumption of full observability, it does *not* use probabilistic programming and does not explicitly represent partial observability. POPKI introduces:

1. **Knowledge state variables** *k*^*t*^(*g*) ∈ {0, 1} for each goal *g* ∈ 𝒢, where *k*^*t*^(*g*) = 1 means the agent *knows* that goal *g* exists.
2. **Perceptual access variable** *o*^*t*^ ∼ *O*(*o* | *s*^*t*^) to model uncertainty in what the agent can see at time *t*. These variables are represented as binary masks over the set of goals, computed from scene geometry and occlusion state.

The causal relationship is straightforward and temporal: perceptual access (*o*^*t*^) drives knowledge accumulation (*k*^*t*^), and knowledge determines which goals the agent can rationally plan toward. An agent that never perceived a goal cannot prefer it; an agent that perceived a goal earlier but currently cannot see it may still act on remembered knowledge. This is precisely the distinction that separates the three trial types in AGENT+.

At a conceptual level, POPKI operates as follows. It treats an observer watching a video as a scientist trying to reverse-engineer the mental state of an agent from its observable behavior. The key insight is that the observer cannot simply read off what the agent knows; they must *infer* it from the physical structure of the scene. A wall between an agent and a reward means the agent could not have seen that reward, and therefore could not have formed a preference about it. POPKI makes this link explicit: it uses the physics engine to determine what was geometrically visible from the agent’s position at each moment, accumulates that information into a belief about what the agent knows, and then uses that belief with a model of rational planning to predict what the agent will do next. When the agent’s actual behavior diverges from this prediction, POPKI registers surprise. The magnitude of that surprise reflects how far the observed action departs from what a rational, informed agent would have done given the inferred knowledge state.

The probabilistic programming implementation in Gen allows POPKI to maintain and update both *k*^*t*^ and *o*^*t*^ as new evidence arrives, enabling principled Bayesian inference over latent mental and physical states. This design enables POPKI to reason about what an agent has **seen** (*o*^*t*^), what it **remembers** (*k*^*t*^), and how this shapes decision-making under uncertainty.

For example, in Type 3 (Figure 1), the familiarization video shows barriers occluding one of the rewards after a brief period of time; POPKI explains this case as the agent having *o*^*t*^(*g*) = 0 (no perceptual access at the end) and relying on *k*^*t*^(*g*) from prior observations. POPKI tracks which goals the agent could see in familiarization (*o*^*t*^) and uses *k*^*t*^ to determine whether unseen but previously encountered goals will still be pursued at test.

### Inference and Surprise Estimation

Given a familiarization video, represented by a trajectory of states *s*^1:*T*^ (Γ_fam_ = *s*^1:*T*^), POPKI performs joint inference over: *k*^1:*T*^, *o*^1:*T*^, Θ = (*R*, *C*), Φ

where:

- *k*^*t*^(*g*) ∈ {0, 1} is the **knowledge state** for goal *g* at time *t*.
- *o*^*t*^(*g*) ∈ {0, 1} is the **stochastic perceptual access** for goal *g* at time *t*.
- Θ = (*R*, *C*) are the agent parameters: *R* is the reward function over goals, *C* is the cost function.
- Φ are the physics parameters of the environment.

The inference is formulated as:

$$P\left(\Phi ,\Theta |\Gamma_{\text{fam}}\right)\propto \sum_{k^{0:T},o^{1:T}}P\left(s^{1:T}|k^{0:T},R,C,\Phi \right)\;P\left(k^{1:T}|o^{1:T},k^{0}\right)\cdot P\left(o^{1:T}|s^{1:T}\right)\;P\left(k^{0}\right)\;P\left(R\right)\;P\left(C\right).$$ (1)

**Figure 2.**
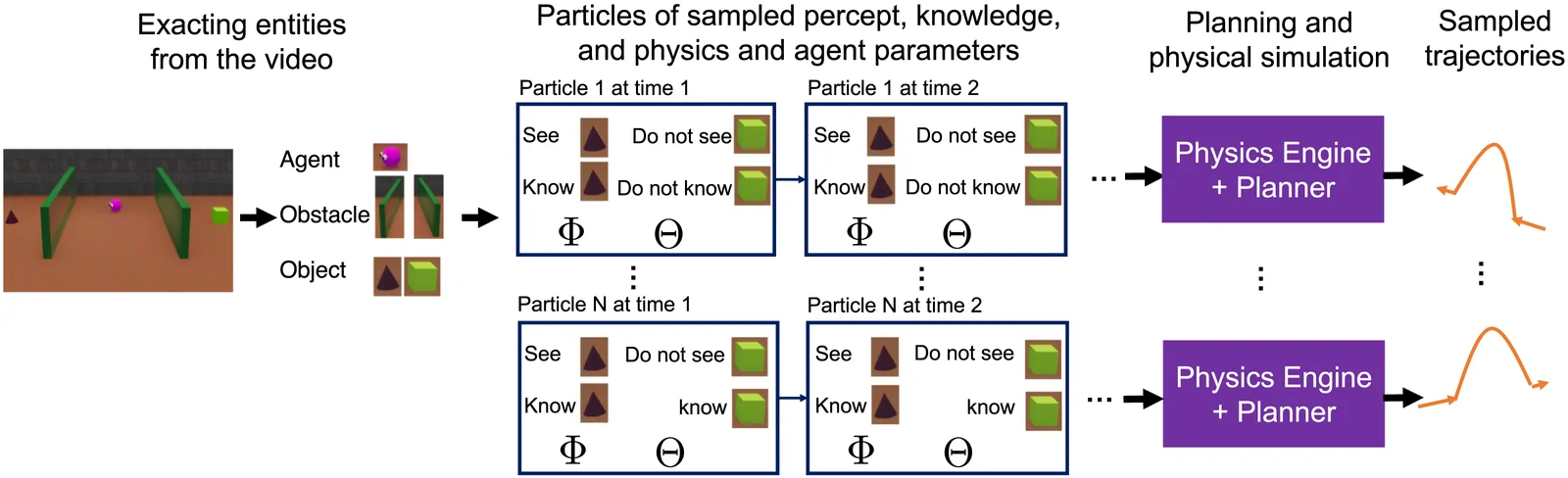
**Overview of the POPKI model.** Each particle represents a joint hypothesis about the agent’s perceptual access history (*o*^1:*T*^), accumulated knowledge state (*k*^1:*T*^), reward function (*R*), cost function (*C*), and physics parameters (Φ). During the *familiarization phase* (top), particles are updated via Sequential Monte Carlo: at each timestep, the physics engine computes which goals are geometrically visible from the agent’s position (*o*^*t*^), updating the knowledge state *k*^*t*^ accordingly. The agent’s trajectory is then simulated under RRT* planning conditioned on *k*^*t*^, and particles are re-weighted by the likelihood of the observed trajectory. The posterior over agent parameters (Φ, Θ) learned from familiarization is then used in the *test phase* (bottom) to compute a surprise rating as the expected log-likelihood of the test trajectory—lower likelihood corresponds to higher surprise.

To compute the likelihood of a trajectory under a given hypothesis, we use a two-step process (Figure 2). First, we simulate the agent’s trajectory in an imagined environment (particle *n*) reconstructed from the hypothesized knowledge state. Then, at each timestep, we compute the likelihood using a Gaussian distribution where the mean is the simulated coordinate at that step and the standard deviation (a constant tuned to match the dataset) allows for small deviations from the predicted path.

We use Sequential Monte Carlo to update knowledge, perceptual access, and preference estimates at each step. Each particle encodes a hypothesis over (*k*^1:*T*^, *o*^1:*T*^, Φ, Θ). We use uniform priors for *R* and *C*, and a Bernoulli prior for *k*^0^ with *P*(knowing an unobserved goal) = 0.5.

For each test video, we compute the **surprise rating** as the expected log-likelihood of observing the agent trajectory in the test environment with respect to the posterior over Φ and Θ from the familiarization phase:

$${𝔼}_{\Phi ,\Theta }\left[\log P\left(\Gamma_{\text{test}}|\Phi ,\Theta \right)\right],$$

where

$$P\left(\Gamma_{\text{test}}|\Phi ,\Theta \right)=\sum_{k^{0:T},o^{1:T}}P\left(s^{1:T}|k^{0:T},R,C,\Phi \right)\cdot P\left(k^{1:T}|o^{1:T},k^{0}\right)\;P\left(o^{1:T}|s^{1:T}\right).$$ (2)

## METHODS

In order to evaluate how humans and models reason about agents’ actions and goals, we designed a set of experiments combining human behavioral data with stimuli from the AGENT+ dataset. Our experiments consisted of (i) collecting human judgments of surprise across a range of trial types, and (ii) computing a standardized evaluation metric that could be applied to both human data and model predictions. Together, these complementary approaches allowed us to directly compare psychological expectations in humans with computational measures of surprise.

### Stimuli

We used videos drawn from the AGENT+ dataset, an extension of the AGENT benchmark for core psychological reasoning. Each video depicts an animated agent navigating an environment containing objects, obstacles, and goal states. The dataset provides multiple modalities (RGB frames, depth maps, and segmentation masks), but in this study, human participants only viewed the rendered RGB videos.

Trials were selected to span various degrees of perceptual access (see Figure 1). In total, we presented 252 test trials. For each surprising condition, a matched expected condition was available, yielding paired comparisons of *expected* versus *surprising* outcomes. Following the dataset design, familiarization videos introduced the agent’s behavior in neutral contexts, while test videos introduced critical events (e.g., detours, obstacle avoidance, or inefficient paths).

### Human Study: Surprise Judgments

We recruited 84 adult participants (mean age = 39.8; 65 female) via Prolific. All participants provided informed consent, and the study was approved by the MIT Institutional Review Board. Participants were randomly assigned to subsets of trials to minimize fatigue while still ensuring that each trial type was evaluated by multiple raters. Each variant was viewed by 10 unique participants, ensuring balanced coverage across conditions. This design resulted in more than 800 human ratings in total.

The experimental procedure consisted of two phases. First, in the *familiarization phase*, participants watched a short set of videos establishing the agent’s typical behavior. Next, in the *test phase*, participants watched a paired sequence of videos: one depicting an expected outcome and one depicting a surprising outcome. After each video, participants rated their level of surprise on a scale from 0 (“not at all surprising”) to 100 (“extremely surprising”). Ratings were self-paced, and participants were encouraged to use the full range of the scale.

### Evaluation Metric

Following Csibra et al. (2003) and Shu et al. (2021), we defined a quantitative metric for surprise. Let *r*^+^ and *r*^−^ denote the mean surprise ratings for the paired surprising and expected test videos, respectively. The *surprise margin* is given by:

$$\Delta r=r^{+}-r^{-}.$$

This margin reflects the relative increase in surprise elicited by a surprising outcome compared to its matched baseline. To make comparisons robust across participants and trial types, ratings were first standardized within participants. Likewise, model outputs were scaled by the number of timesteps within each video so that the computed margins were not biased by scale differences across conditions.

By applying the same metric to both human data and model predictions, we obtained a common ground for evaluating whether models captured the same qualitative patterns of psychological reasoning as humans.

## BASELINES

### ToMnet-G (Modified)

**Figure 3.**
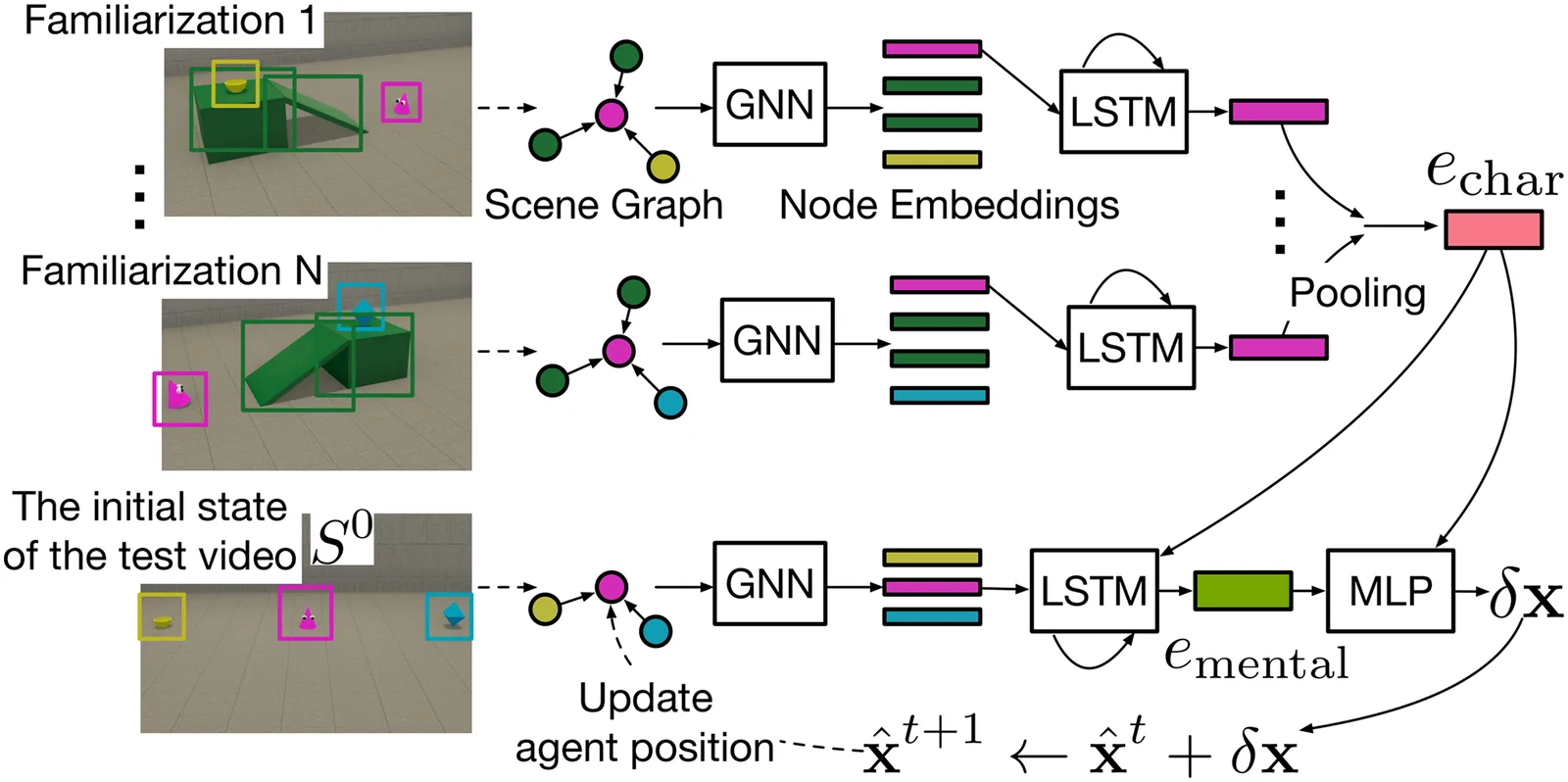
**Structure of ToMnet-G (modified).** Scene graphs are constructed from ground-truth entity positions and visibility masks, then processed by a graph neural network (GNN) to produce a character embedding *e*_char_ from familiarization videos. This embedding is combined with test-video scene features and passed through an LSTM to predict the agent’s next displacement *δx*. Unlike POPKI, ToMnet-G has no explicit representation of the agent’s belief state or perceptual history; visibility constraints are encoded implicitly through the input features.

ToMnet-G (Rabinowitz et al., 2018; Shu et al., 2021) is a deep learning model that uses graph neural networks (GNNs) to infer an agent’s mental states (Figure 3). Entities such as objects and agents are represented as nodes with attributes like position and color, while edges capture spatial and relational structure.

To adapt ToMnet-G to the AGENT+ dataset, we modified the original model to include partial perceptual access: the familiarization embedding is conditioned not only on entity positions, but also on visibility constraints for each timestep. This update enables the model to learn from perceptual occlusion patterns during training, a crucial addition for fairness against POPKI (Appendix 1B).

ToMnet-G is trained in an end-to-end manner, where the prediction of agent behavior is learned directly from input scenes and trajectories without an explicit simulation of the agent’s decision-making process. Despite its expressiveness, it lacks explicit modeling of physical reasoning or persistent memory for prior perceptual states, which limits its performance in tasks that require long-term belief tracking.

### Vision-Language Models

We tested Google Gemini 2.5 Pro and OpenAI GPT-4o. Given keyframes and a question prompt, these VLMs showed weak or no correlation with human surprise judgments (Gemini: *r* = −0.037, *p* = 0.86; GPT-4o: *r* = 0.19, *p* = 0.36; full results in Model Comparison section). While powerful in commonsense reasoning and visual QA, VLMs lack mechanisms to model agent-specific knowledge or past visual access, and thus fail to infer belief divergence due to occlusion.

These models are capable of open-ended visual and image reasoning, and excel at integrating natural language with perception. However, they are not designed to track agent-specific beliefs over time or simulate knowledge divergence due to occlusion. While VLMs can describe scenes and infer intentions from static frames, they do not infer what the agent did or did not see based on temporal occlusion, leading to systematic failures in the AGENT+ scenarios.

## RESULTS AND DISCUSSION

### Human Behavior

**Figure 4.**
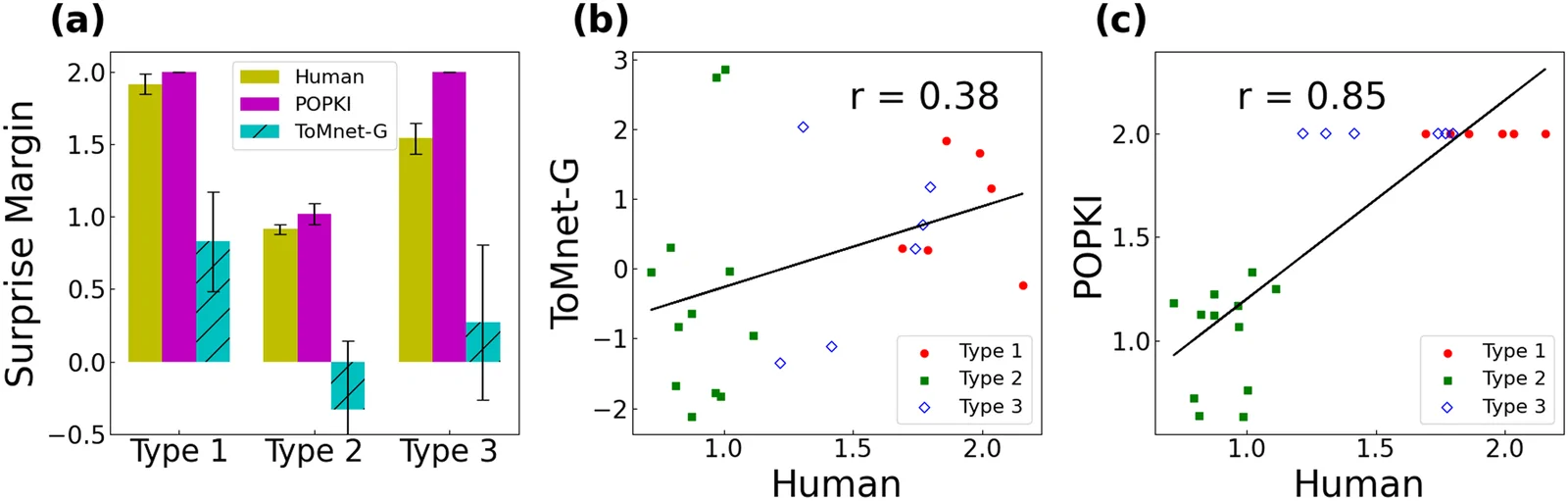
**Surprise margin comparisons between humans and models.** (a) Average surprise margins for human participants and each model across the three trial types. Human data (yellow) show high margins for Types 1 and 3 and a lower margin for Type 2, reflecting sensitivity to the agent’s perceptual access history. POPKI (magenta) closely tracks this pattern; ToMnet-G (blue) and VLMs (not shown) do not. (b) Scatter plot of trial-level surprise margins: human participants vs. ToMnet-G (*r* = 0.38, *p* = 0.07); no significant correlation. (c) Scatter plot of trial-level surprise margins: human participants vs. POPKI (*r* = 0.85, *p* < 0.001); strong correlation across all trial types.

Human participants demonstrated distinct patterns in surprise ratings depending on the trial type. As expected, participants’ ratings varied systematically across trial types: surprise was highest in Type 1 and Type 3 conditions, which involved deviations in the test phase from the familiarization phase, and lowest in Type 2 limited perceptual access conditions (Figure 4). A one-way ANOVA revealed significant differences in human surprise scores across trial types (*F*(2, 252) = 84.616, *p* < 0.001, *η*^2^ = 0.890). In both Type 1 (equal perceptual access) and Type 3 (initial access with occlusion), participants rated the “surprising” test videos as significantly more surprising than their expected counterparts (surprise rating [mean/standard deviation] of [1.92/0.07] and [1.54/0.11], respectively). When averaged across participants, these differences were robust and consistent—achieving near 100% agreement at the group level.

By contrast, Type 2 trials (limited perceptual access) yielded more ambiguous responses. Surprise ratings for expected vs. surprising videos in this condition were far closer, clustering around the midpoint of the scale (a surprise rating of 1.0). This suggests that participants appropriately adjusted their expectations based on what the agent had seen. Because the agent lacked perceptual access to one object during familiarization, its prior behavior does not constitute clear evidence of preference and the choice to approach the newly visible object at test could itself be entirely rational and optimal, reflecting a genuine preference that the agent simply had no opportunity to express before. Participants therefore withheld strong surprise in either test condition, recognizing that the agent’s constrained history left its true preference underdetermined.

Interestingly, Type 3 trials received roughly 20% lower surprise ratings than Type 1, indicating that participants took into account the agent’s memory of earlier perceptual access, but with some uncertainty. This subtle gradient in surprise responses underscores the complexity of human belief tracking under perceptual uncertainty.

### Model Comparison

#### POPKI.

POPKI replicated the key qualitative patterns observed in human data. The model produced large and stable surprise margins for Types 1 and 3, and lower, more variable margins for Type 2, broadly aligning with human responses. Similarly, a one-way ANOVA also revealed significant differences in model surprise scores across trial types (*F*(2, 252) = 74.610, *p* < 0.001, *η*^2^ = 0.877).

On average, POPKI’s surprise margins for Types 1 and 3 were approximately 2.0, consistent with human-derived margins and yielding a positive Pearson’s correlation (*r* = 0.85, *p* < 0.001). The model’s correlation with human surprise ratings was strong across all trial types, including the memory-based Type 3 condition that required reasoning about earlier perceptual access.

Importantly, POPKI’s probabilistic treatment of knowledge and perceptual access allowed it to produce graded surprise scores—rather than binary expectation violations—capturing subtle shifts in inference when the agent operated under occlusion or partial knowledge. This mechanism enabled the model to distinguish between trial Types 1 and 2, as well as between Types 3 and 2, in a manner consistent with human behavior.

However, as shown in Figure 4c, POPKI does not fully capture the slight and insignificant difference in human ratings between Types 1 and 3: humans rated Type 3 scenarios considerably *less* surprising than Type 1, suggesting that people incorporate an additional degree of uncertainty about the agent’s memory that is not fully modeled in POPKI’s current formulation (Figure 4a). This residual gap indicates a potential avenue for refinement, such as incorporating graded memory decay or variable confidence in retained knowledge states. Nonetheless, the calibrated surprise margin computation enhances POPKI’s stability and predictive accuracy across the full range of trial types.

#### ToMnet-G.

In contrast, ToMnet-G failed to capture the structure of surprise across perceptual access conditions. Although the model was adapted to encode visibility constraints, its predictions did not significantly correlate with human judgments (*r* = 0.38, *p* = 0.07), and performance did not improve even under different generalization regimes (e.g., training on different combinations of trial types).

Even when ToMnet-G was trained with leave-one-type-out protocols or evaluated on perceptual access trials exclusively, it struggled to differentiate between surprising and expected conditions in a consistent, human-like manner. Its surprise margins were weak and showed little variation between trial types.

#### Vision-Language Models (VLMs).

State-of-the-art VLMs such as Google Gemini 2.5 Pro and OpenAI GPT-4o performed poorly on this task. Despite receiving the same familiarization/test keyframes and natural language prompts as human participants, the models’ surprise ratings showed either no Pearson’s correlation (Gemini: *r* = −0.037, *p* = 0.86) or only weak, non-significant correlation (GPT-4o: *r* = 0.19, *p* = 0.36) with human data.

These results reveal a major gap in the ability of even advanced multimodal models to perform perspective-taking or simulate partial observability. Without explicit representations of what the agent can or cannot see—and how this changes over time—VLMs cannot infer mental states that diverge from their own “omniscient” view of the scene.

## GENERAL DISCUSSION

We have introduced AGENT+, a novel benchmark for evaluating ToM reasoning in physically grounded scenarios with partial perceptual access. Through a series of controlled experiments, we demonstrated that humans make systematic and robust inferences about agent beliefs and preferences based on prior visibility, and that their judgments vary depending on whether perceptual access was equal, partial, or temporally restricted.

To model this behavior, we proposed POPKI, a generative Bayesian framework that integrates perceptual access, agent knowledge, and physical simulation within a unified inverse planning system. Unlike end-to-end baselines such as ToMnet-G or open-ended VLMs like Gemini and GPT-4o, POPKI explicitly tracks what the agent has seen over time and simulates its decision-making accordingly. This structure allowed POPKI to closely match human surprise patterns across all trial types, especially in scenarios where belief divergence emerges from occlusion.

Our findings emphasize the importance of modeling perceptual access in agent-centric reasoning. While current deep learning models can capture scene statistics or make coarse predictions about behavior, they lack a structured representation of agents’ informational boundaries. POPKI’s ability to infer graded beliefs and simulate rational planning under perceptual constraints marks a key advance in cognitively informed AI.

Moreover, our findings reveal that humans exhibit slightly lower, but insignificant, surprise levels when an agent has knowledge without perceptual access—a distinction not anticipated by existing models such as POPKI (Figure 4c). Extending POPKI to account for such working memory effects could deepen the predictive accuracy of computational models of cognition.

Future work should explore integrating POPKI-like inference with large-scale visual representations, extending the benchmark to multi-agent social reasoning, and accounting for the aforementioned memory and decay in human judgments. By bridging structured modeling with real-world perception, we take a step toward artificial agents that reason about others as humans do—in a world where knowledge is partial, and perception is likewise constrained.

## FUNDING INFORMATION

This work was supported by the DARPA Machine Common Sense program, MIT-IBM AI LAB, and NSF STC award CCF-1231216.

## APPENDIX 1A: GPT-4O PROMPTS

We use GPT-4o to provide a surprise rating in response to key frames from a video. The prompts are provided as follows.

Code Example {“role”: “system”, “content”: ( “You analyze test videos based on how the agent behaved in a prior familiarization video.” “The agent is a colored block with two eyes in the center of the frame.” “Based on its past behavior in the familiarization video, assess how surprising its actions are in the test video.” )}, {“role”: “user”, “content”: [{“type”: “text”, “text”: ( “Below are frames from a familiarization video followed by a test video.” “Rate how surprising the agent’s behavior in the test video is, given what it did in the familiarization video.” “Give a numeric surprise score between 0 (not surprising at all) and 100 (very surprising), and explain your reasoning.” )}, *image_parts ]}

## APPENDIX 1B: GEMINI 2.5 PRO PROMPTS

We use Google Gemini 2.5 Pro (Preview 3–25) to provide a surprise rating in response to key frames from a video. The prompts are provided as follows.

Code Example You are an AI trained to analyze visual sequences in the context of agent behavior and expectations. You will be shown frames from three short videos: Familiarization Video: This shows how the agent (a colored block with two “eyes”) typically behaves—especially which objects it moves toward or avoids. Test Video A: This shows the agent in a new situation. Your task is to assess how surprising the agent’s behavior is compared to the familiarization video. Does it behave as expected based on what it did in the expected video? Test Video B: Another new situation. Again, judge how surprising the agent’s actions are compared to what you observed in the familiarization video. The agent is typically positioned in the center of the scene and makes a choice by moving closer to one of two objects (reward options). Your task is to assign a surprise score from 0 to 100 for each test video: A score of 0 means the agent behaves exactly as expected based on the familiarization video. A score of 100 means the behavior is completely unexpected or extremely surprising. Be sure to justify each score in 2 to 3 sentences by describing the agent’s decision in the test video and how it compares to the familiarization video.

## APPENDIX 1C: TOMNET-G

### Network Architecture

Each GNN consists of *N* nodes; each node has an input, *v*_*i*_ = (*x*_*i*_, *a*_*i*_), where *x*_*i*_ is the center of the 3D bounding box *x*_*i*_, and *a*_*i*_ is the appearance information, including the object label, the width, length, and height of the 3D bounding box, and the orientation of the bounding box. The node input is encoded by *ϕ*_*v*_(*v*_*i*_) = [$\phi_{v}^{x}$(*x*_*i*_), $\phi_{v}^{a}$(*a*_*i*_)], where both $\phi_{v}^{x}$ and $\phi_{v}^{a}$ are a 32-dim fully-connected (FC) layer. The edge between node *i* and *j* is encoded by a 64-dim FC layer, *ϕ*_*e*_(*v*_*i*_, *v*_*j*_). For the agent node, we connect all other entities to it and aggregate the edge embeddings by a sum-pool. Concatenated with the embedding of the agent node itself, we get

$$\left[\phi_{v}\left(v_{i}\right),\sum_{j\neq i}\phi_{e}\left(v_{i},v_{j}\right)\right],$$

which is passed to a 64-dim FC layer to get the final agent node embedding

$$\phi_{\text{agent}}\left(\left[\phi_{v}\left(v_{i}\right),\sum_{j\neq i}\phi_{e}\left(v_{i},v_{j}\right)\right]\right).$$

For a familiarization video *k*, this is then passed to an LSTM with 64 hidden units to get an encoding of the video $e_{\text{fam}}^{k}$ (i.e., the last latent state of the LSTM). By a sum-pool over the encoding of all familiarization videos, we get

$$e_{\text{char}}=\sum_{k}e_{\text{fam}}^{k}.$$

For a test video, the agent node embedding is concatenated with *e*_char_ and passed to two 64-dim FC layers, followed by an LSTM with 64 hidden units. The hidden state of this LSTM becomes *e*_mental_. Concatenating *e*_mental_ and *e*_char_, we predict the displacement of the agent in one step *δx* with an FC layer. *δx* is then used to update *x*_*i*_ in the agent node’s input. We predict the movement of the agent until the end of the test video.

### Training

We train the network using Adam (Kingma & Ba, 2017), with a batch size of 16, and a learning rate of 0.001.
